# Effect of MEFV mutations and HLA-B27 on clinical findings of familial Mediterranean fever and spondyloarthritis

**DOI:** 10.1097/MD.0000000000045344

**Published:** 2025-10-24

**Authors:** Tuba Yuce Inel, Aslihan Avanoglu Guler, Nazife Sule Yasar Bilge, Timucin Kasifoglu, Abdurrahman Tufan, Ismail Sari

**Affiliations:** a Division of Rheumatology, Dokuz Eylul University, Izmir, Turkey; b Division of Rheumatology, Faculty of Medicine, Gazi University, Ankara, Turkey; c Division of Rheumatology, Faculty of Medicine, Eskisehir Osmangazi University, Eskisehir, Turkey.

**Keywords:** amyloidosis, ankylosing spondylitis, familial Mediterranean fever, HLA-B27, MEFV, spondyloarthritis

## Abstract

This study investigated the coexistence of familial Mediterranean fever (FMF) and spondyloarthritis (SpA), aiming to provide a comprehensive understanding of their clinical, radiological, and genetic features. A total of 127 patients with both FMF and SpA were evaluated, with data collected on demographic, clinical, and laboratory characteristics, including human leukocyte antigen (HLA)-B27 status, Mediterranean fever (MEFV) gene variants, and acute phase reactants. Pelvic radiographs and sacroiliac joint magnetic resonance imaging scans were reviewed when available. The median age of the cohort was 39 years (interquartile range: 30–50), and 48% were female, showing an equal sex distribution. Ankylosing spondylitis was the most common SpA subtype (76.4%), followed by nonradiographic axial SpA (10.2%), peripheral SpA (5.5%), undifferentiated SpA (6.3%), and psoriatic arthritis (1.6%). Most patients exhibited intermittent (90.8%) and oligoarticular (76.7%) joint involvement, typically affecting large joints of the lower extremities, while 23.6% had chronic arthritis. Patients with grade 4 sacroiliitis, chronic arthritis, or hip arthroplasty had a longer disease duration (*P* < .05). HLA-B27 positivity was identified in 30.4% of patients and was strongly associated with AA amyloidosis (odds ratio = 13.3, 95% confidence interval: 4–43.9). The most frequent MEFV mutation was homozygous M694V (69.3%), followed by M694V/M680I (14.7%) and M680I/V726A (5.3%). MEFV mutations were present in 83.1% of HLA-B27-negative patients. Collectively, these findings indicate that FMF-SpA coexistence defines a distinct clinical and genetic entity characterized by balanced sex distribution, lower HLA-B27 frequency compared to classic SpA, and a strong link between HLA-B27 positivity and amyloidosis risk, underscoring a complex genetic interaction between MEFV variants and HLA-B27 in disease expression.

**Key Points**

• In patients with FMF, the presence of inflammatory back pain, psoriasis, uveitis, inflammatory bowel disease, or enthesitis alongside chronic large joint arthritis suggests possible underlying SpA.

• Testing for HLA-B27 and MEFV genetic mutations is essential not only for diagnosis but also for assessing prognosis.

• The likelihood of developing amyloidosis is notably higher in patients who are HLA-B27 positive and carry the M694V mutation, making regular monitoring of proteinuria important in this group.

• Moderate to severe hip joint involvement and the need for prosthetics are significantly elevated in those with the M694V mutation. Consequently, early radiological screening for these patients is crucial in preserving physical function.

## 1. Introduction

Familial Mediterranean fever (FMF) is a hereditary autoinflammatory disease characterized by recurrent, self-limiting, febrile serosal and musculoskeletal inflammatory attacks.^[1,2]^ Mutations in the Mediterranean fever (MEFV) gene encoding pyrin, a key regulator of inflammatory pathways, result in clinical symptoms due to overproduction of IL-1 (1, 2).

MEFV mutations have emerged as significant contributors to the pathogenesis of spondyloarthritis (SpA), including ankylosing spondylitis (AS), independent of the clinical manifestations associated with FMF.^[3]^ Research has highlighted a noteworthy increase in the prevalence of the M694V variant among AS patients without FMF symptoms, particularly when compared to healthy individuals.^[4,5]^ Additionally, IL-1 promotes Th17 differentiation,^[6,7]^ and serum amyloid A activates the IL-17 pathway.^[8]^ Genetic and clinical studies have shown that the IL-17 pathway has an essential role in the pathogenesis of SpA.^[9]^ Therefore, MEFV mutations may contribute to SpA-related clinical findings through IL-1 induction of the IL-17 pathway.

Several studies have indicated an increased relationship between SpA and FMF.^[10–13]^ Notably, both diseases exhibit clinical and pathogenetic similarities, such as early onset, large joint arthritis predominantly affecting the lower extremities, hip joint involvement, and the need for hip joint arthroplasty in some cases.^[1,13]^ A subset of FMF patients present with sacroiliitis and enthesitis.^[10,14]^ Additionally, there is a significant rise in the frequency of SpA observed in first-degree relatives of FMF patients.^[15]^

Given the limited number of reports and the need for further data, this study aims to comprehensively demonstrate the demographic, clinical, and radiological characteristics of patients with FMF and SpA. By investigating these characteristics, the study aims to provide a clearer understanding of the relationship between FMF and SpA, which may inform future research and clinical practice.

## 2. Methods

This multi-center, retrospective, descriptive study involved patients diagnosed with FMF and SpA from the Rheumatology departments at Dokuz Eylul University, Gazi University, and Eskisehir Osmangazi University. All patients with regular follow-up data since 2010 were included in the study. To confirm the preexisting clinical diagnoses of FMF and SpA in the participating patients, Tel-Hashomer^[16]^ and Assessment of SpondyloArthritis International Society (ASAS) criteria^[17,18]^ were used, along with Amor,^[19]^ and European Spondyloarthropathy Study Group criteria^[20]^ for broader inclusion of SpA manifestations. Patients who exhibited SpA symptoms but were not classified as SpA by ASAS criteria were also recorded.

Detailed patient data were collected, including age at FMF diagnosis and specific FMF symptoms such as peritonitis, pleuritis, arthritis, and fever. The medical records of patients diagnosed with FMF in childhood were also examined. If records were unavailable, patient anamnesis was used to identify FMF symptoms that began in childhood. For SpA, data encompassed age at SpA diagnosis and SpA symptoms such as inflammatory back pain (based on Calin criteria),^[21]^ arthritis, and enthesitis. Patients with SpA were further classified based on the dominant involvement type (axial or peripheral). Additionally, treatment approaches, family history of SpA and FMF, and complications such as AA amyloidosis and joint arthroplasty were documented. Patients were treated according to European League Against Rheumatism (EULAR) recommendations.

Laboratory findings, if available in medical records, included human leukocyte antigen (HLA)-B27, MEFV gene analysis, and acute phase reactants at the time of diagnosis and the last visit. Cervical and lumbar radiographs were evaluated in terms of syndesmophytes, and the sacroiliac joints were assessed using the modified New York (mNY) criteria.^[22]^ Radiographic hip joint involvement was evaluated using the BASRI-hip score.^[23]^

This study received ethical approval from the Dokuz Eylul University Faculty of Medicine, noninvasive Clinical Ethics Committee (Decision No: 2020/21-09), and all procedures strictly adhered to the ethical principles of the Declaration of Helsinki. Written informed consent was obtained from all patients.

### 2.1. Statistical analysis

Data entry and analysis were executed utilizing SPSS 16. The assessment of normality was carried out through the Kolmogorov–Smirnov test. Median values accompanied by their respective minimum-maximum ranges were used to present continuous variables, while percentages were utilized for nominal and ordinal data. To compare 2 independent groups, the Mann–Whitney *U* test was employed for continuous variables, and Fisher exact test was utilized for categorical variables. To identify predictive factors influencing various outcomes of FMF and SpA, binary logistic regression analysis was applied. Statistical significance was established at a two-tailed *P* value of .05 or lower.

## 3. Results

### 3.1. Clinical and demographic characteristics

The study enrolled 127 patients with coexistent FMF-SpA, with a median age of 39 years (interquartile range [IQR]: 30–50). Of these patients, 66 were male (52%). The median age at diagnosis for FMF patients was 25 years (IQR: 17–36), and the median duration of diagnosis was 12 years (IQR: 8–17). Conversely, the median age at diagnosis for SpA was 28 years (IQR: 21–37), and the median duration of diagnosis was 9 years (IQR: 5–15). FMF symptoms preceded SpA in 47.2% (n = 60) of cases, while SpA symptoms appeared before FMF in 27.6%.

FMF manifestations included abdominal pain (88.2%), fever (81.7%), pleuritic chest pain (54.8%), erysipelas-like erythema (37.6%), and myalgia (25.8%). The history of arthritis was apparent in 73.2% of patients, with 78% having large joint involvement, 11% affecting both large and small joints, and 11% exclusively involving small joints. Arthritis predominantly exhibited an oligo-articular pattern in 76.7% of patients, while 13.3% presented with a mono-articular type, and 10% demonstrated a polyarticular type. The arthritis pattern was intermittent in 90.8%, migratory in 6.9%, and additive in 2.3%. Chronic arthritis was observed in 23.8% of patients. Histologically proven AA amyloidosis was diagnosed in 10.3% (n = 13) of patients.

In a total of 104 patients (81.9%), axial symptoms were the dominant symptom type. Most patients were classified as ASAS axSpA (n = 110, 86.6%). Of these, the majority (n = 97, 88.2%) had radiographic axSpA (also known as AS), while the remaining 13 (11.8%) had non-radiographic axSpA. A smaller group of patients (8.1%) were classified as ASAS peripheral SpA. Additionally, 6.3% of patients did not meet the ASAS classification but displayed characteristic SpA features such as psoriasis, uveitis, and chronic inflammatory back pain. Regarding previous SpA classification criteria, 97 patients (76.4%) met the Amor criteria, and 112 (88.2%) met the European Spondyloarthropathy Study Group criteria.

In the overall group, various SpA-related characteristics were identified: enthesitis was present in 18.1% of patients, dactylitis in 3.9%, uveitis in 7.1%, psoriasis in 4.7%, and inflammatory bowel disease in 8.7% of cases. Within the cohort, sacroiliac magnetic resonance imagings were available for 76 patients (59.8%), with 68.4% of these showing bone marrow edema in line with ASAS definitions. Furthermore, 97 patients (76.4%) had cervical lateral X-rays, and 113 patients (89%) had lumbar lateral X-rays taken within 2 years of their last visit. Among these individuals, 21.6% displayed cervical syndesmophytes, while lumbar syndesmophytes were observed in 36.3% of patients. Among the 80 patients who had both cervical and lumbar X-rays, 22.5% exhibited syndesmophytes in both regions, while 77.5% had no syndesmophytes in either cervical or lumbar areas. Radiologically, moderate to severe hip involvement was evident in 31 patients (25.6%), and 11 patients (8.7%) had total hip joint replacement.

Regarding family history, 52 patients (41.6%) had first-degree family members diagnosed with FMF, and 20 patients (16%) had second-degree relatives affected by FMF. In relation to SpA, 30 patients (26.3%) had first-degree relatives with SpA, and 4 patients (3.5%) had second-degree relatives affected. A concise summary of the demographic and clinical characteristics of the patients can be found in Table 1.

**Table 1:** Clinical and demographic characteristics of the patients, n (%).

Age*	39 (30–50)
Sex (male)	66 (52)
Age at diagnosis of FMF*	25 (17–36)
FMF diagnosis duration*	12 (8–17)
FMF clinical findings	
Fever	104 (81.9)
Peritonitis	111 (87.4)
Pleuritis	67 (52.8)
Erysipelas-like rash	39 (30.7)
Standing myalgia	29 (22.8)
Arthritis patterns	
Intermittent	79 (90.8)
Additive	2 (2.3)
Migratory	6 (6.9)
Arthritis type	
Monoarthritis	12 (13.3)
Oligoarthritis	69 (76.7)
Polyarthritis	9 (10)
Joint involvement	
Large joint	71 (78)
Large and small joint	10 (11)
Small joint	10 (11)
Chronic arthritis	30 (24)
Age at diagnosis of SpA*	28 (21–37)
SpA diagnosis duration*	9 (5–15)
SpA subgroup	
Ankylosing spondylitis	97 (76.4)
Non-radiographic axial SpA	13 (10.2)
Peripheral SpA	7 (5.5)
Undifferentiated SpA	8 (6.3)
PsA	2 (1.6)
The current dominant SpA type	
Axial dominant	104 (81.9)
Peripheral dominant	23 (18.1)
SpA clinical findings	
Inflammatory back pain	109 (85.8)
Psoriasis	6 (4.7)
Dactylitis	5 (3.9)
Enthesitis	23 (18.1)
Uveitis	9 (7.1)
Inflammatory bowel disease	11 (8.7)
HLA-B27 positivity	31 (30.4)
Any syndesmophyte	44 (38.9)
Cervical and lumbar syndesmophyte	18 (18.6)
Coxofemoral joint involvement	31 (25.6)
Hip prosthesis	11 (8.7)
FMF family history	
1st degree	52 (41.6)
2 degrees	20 (16)
Family history of SpA	
1st degree	30 (26.3)
2 degrees	4 (3.5)
Amyloidosis	13 (10.3)
Family history of amyloidosis	12 (9.5)
Treatment	
Biological agents at any time	72 (56.7)
IL-1 inhibitors at any time	25 (19.7)
Non-IL-1 biological agents at any time	65 (51.2)

FMF = familial Mediterranean fever, HLA = human leukocyte antigen, SpA = spondyloarthritis.

* Years, median (IQR).

### 3.2. Genotype and allele distributions

We conducted an analysis of genotype and allele distributions among a cohort of 106 patients, which accounted for 83.5% of the total population, as they had available MEFV data. Remarkably, all but one patient exhibited at least one documented mutation. Seventy-five (73.5%) of patients had either homozygous or compound heterozygous pathogenic exon 10 MEFV variants, while 27 patients (26.4%) displayed heterozygous exon 10 variants. Homozygous M694V was the most prevalent variant (n = 52, 51%), followed by heterozygous M694V (n = 19, 18.6%), M694V/M680I (n = 11, 10.8%), M680I/V726A (n = 4, 3.9%), and V726A heterozygous (n = 4, 3.9%) variants. The overall carrier rate of M694V and M680I mutations was 85.2% (n = 87) and 19% (n = 20) respectively.

In addition to genotypic variations, we conducted a separate examination of mutation allele frequencies. The results indicated that the M694V variant was the most prevalent at 60.4% (n = 139), followed by the M680I variant at 10% (n = 23) and V726A at 4.8% (n = 11). For a detailed analysis of genotype and allelic distributions, please refer to Table S1 (Supplemental Digital Content, https://links.lww.com/MD/Q402).

#### 3.2.1. Impact of MEFV mutations on clinical phenotype

We explored the potential associations between MEFV variants and clinical characteristics of patients. We did not find a significant association between the type of MEFV variants and the presence, type and pattern of arthritis, structural changes like the presence of syndesmophytes in both cervical and lumbar regions. However, MEFV mutation status was significantly linked to joint replacement surgery (Table S2, Supplemental Digital Content, https://links.lww.com/MD/Q402). On the other hand, the presence of any M694V allele was significantly associated with the occurrence of amyloidosis. Furthermore, M694V was related to erysipelas-like erythema, chronic arthritis, large joint arthritis, arthroplasty, and a trend toward the absence of dactylitis (Table 2).

**Table 2:** The effect of M694V mutation on clinical findings of FMF and SpA.

	M694V mutation (+) n (%)	M694V mutation (-) n (%)	*P*-value
Gender (Male)	75 (54)	43 (47.3)	.32
FMF clinical findings			
Fever	116 (83.5)	78 (85.7)	.64
Peritonitis	117 (84.2)	79 (86.8)	.58
Pleuritis	74 (53.2)	48 (52.7)	.94
Erysipelas-like rash	55 (39.6)	23 (25.3)	.03
Febrile myalgia	37 (26.6)	21 (23.1)	.55
SpA diagnosis duration, yr	10.1 (±7.36)	10.45 (±9.71)	.80
The current dominant SpA clinic			
Peripheral dominant	24 (17.3)	22 (24.2)	.24
Axial dominant	115 (82.7)	69 (75.8)	
SpA clinical findings			
Inflammatory back pain	119 (85.6)	75 (82.4)	.51
Enthesitis	23 (16.5)	17 (18.7)	.68
Dactylitis	2 (1.4)	6 (6.6)	.06
Uveitis	9 (6.5)	5 (5.5)	.76
Inflammatory bowel disease	14 (10.2)	6 (6.6)	.34
Psoriasis	3 (2.2)	3 (3.3)	.45
Chronic arthritis	47 (34.3)	17 (18.7)	.01
Arthritis type			
Monoarthritis	16 (16.7)	8 (14.8)	.77
Oligoarthritis	80 (83.3)	46 (85.2)	
Arthritis pattern			
Intermittent	90 (89.1)	56 (88.9)	.25
Additive	1 (1)	3 (4.8)	
Migratory	10 (9.9)	4 (6.3)	
HLA-B27 positivity	31 (27.4)	21 (28.8)	.84
Amyloidosis	21 (15.1)	3 (3.3)	.004
Any syndesmophyte	56 (46.3)	28 (34.6)	.1
Both cervical + lumbar syndesmophyte	22 (19.8)	14 (18.7)	.85
Moderate to severe hip joint involvement	46 (35.1)	18 (20.7)	.02
Hip prosthesis	18 (12.9)	4 (4.4)	.03

Please be aware that the analysis was performed within the group of tested patients. As a result, the numerical outcomes deviate from the actual counts based on the number of individuals. This discrepancy arises because the numbers presented represent allelic counts, not individual counts.

FMF = familial Mediterranean fever, HLA = human leukocyte antigen, SpA = spondyloarthritis.

### 3.3. Impact of HLA-B27 on clinical phenotype

HLA-B27 data was available in 105 patients, and HLA-B27 was positive in 31 (30.4%) of the patients. Upon analyzing groups based on HLA-B27 presence, it was observed that HLA-B27 positivity showed a higher prevalence within the axial dominant subtype among males, those with chronic arthritis, and individuals with a greater occurrence of amyloidosis (Table 3).

**Table 3:** The effect of HLA-B27 on clinical findings of FMF and SpA.

	HLA-B27 (+) n (%)	HLA-B27 (-) n (%)	*P*-value
Gender (Male)	20 (64.5)	35 (49.3)	.16
FMF clinical findings			
Fever	24 (77.4)	59 (83.1)	.5
Peritonitis	28 (90.3)	60 (84.5)	.33
Pleuritis	12 (38.7)	41 (57.7)	.08
Erysipelas-like rash	8 (25.8)	23 (32.4)	.51
Febrile myalgia	5 (16.1)	19 (26.8)	.24
Age at diagnosis of SpA*	26.5 (±14.4)	30.1 (±10.7)	.16
SpA diagnosis duration*	15.3 (±8.9)	8.7 (±7.01)	.001
The current dominant SpA clinic			
Peripheral dominant	3 (9.7)	14 (19.7)	.21
Axial dominant	28 (90.3)	57 (80.3)	
SpA clinical findings			
Inflammatory back pain	26 (83.9)	65 (91.5)	.21
Enthesitis	4 (12.9)	15 (21.1)	.33
Dactylitis	0 (0)	3 (4.2)	.33
Uveitis	3 (9.7)	4 (5.6)	.36
Inflammatory bowel disease	3 (10.0)	7 (9.9)	.62
Psoriasis	0 (0)	4 (5.6)	.23
Chronic arthritis	11 (35.5)	14 (20.3)	.11
Arthritis type			
Monoarthritis	4 (21.1)	6 (14.3)	.38
Oligoarthritis	15 (78.9)	36 (85.7)	
Arthritis pattern			
Intermittent	20 (90.9)	41 (89.1)	.73
Additive	1 (4.5)	1 (2.2)	
Migratory	1 (4.5)	4 (8.7)	
Amyloidosis	8 (26.7)	2 (2.8)	.001
Any MEFV mutation	26 (83.9)	59 (83.1)	.8
Any syndesmophyte	13 (43.3)	21 (34.4)	.41
Cervical + lumbar syndesmophyte	7 (24.1)	9 (17.0)	.43
Moderate to severe hip joint involvement	11 (36.7)	16 (23.9)	.19
Hip prosthesis	3 (9.7)	6 (8.5)	.55

FMF = familial Mediterranean fever, HLA = human leukocyte antigen, MEFV = Mediterranean fever, SpA = spondyloarthritis.

* Years, ±SD.

### 3.4. Multivariate analysis

In our study, we examined the influence of specific genotypes, namely HLA-B27 and M694V, on several clinically relevant phenotypes. In the univariate model, our findings indicated that HLA-B27 holds substantial predictive power for amyloidosis, as evidenced by an odds ratio (OR) of 13.8 (95% CI: 3.8–49.7). Similarly, the M694V mutation emerged as a noteworthy predictor of amyloidosis, with an OR of 5.2 (95% CI: 1.5–18). However, when both variables were simultaneously introduced into the model, it became apparent that HLA-B27 retained its predictive significance, yielding an OR of 10.6 (95% CI: 2.8–40.5) for amyloidosis. On the other hand, concerning the requirement of arthroplasty, the M694V variant displayed significance as a predictor, boasting an OR of 3.2 (95% CI: 1.1–9.8), while HLA-B27 did not exhibit a significant predictive role in this context. It’s worth noting that neither of these factors was predictive of the presence of syndesmophytes.

## 4. Discussion

Based on a thorough evaluation, our study stands as one of the largest cohorts of individuals with both FMF and SpA, yielding pivotal insights into their characteristics. The findings underscore the following key revelations: Gender distribution is notably balanced, demonstrating an equal impact of FMF and SpA across both sexes; A temporal sequence is evident, with FMF symptoms preceding the onset of SpA symptoms in 47.2% of cases; The shared symptom of arthritis takes the form of an intermittent pattern, primarily affecting large joints in an oligo-articular manner, frequently involving the lower extremities; The predominant diagnosis among the cohort is radiographic axSpA, commonly referred to as AS; Intriguingly, a significant subset of patients does not exhibit radiographically evident spine damage, highlighting the spectrum of disease severity; The prevalence of HLA-B27, often linked with axSpA, is notably lower in this cohort compared to classical axSpA groups; A remarkable finding surfaces: HLA-B27’s role in contributing to amyloidosis is more pronounced when both HLA-B27 and M694V coexist, accentuating genetic complexities; and Notably, the prediction of hip joint arthritis leans more towards the M694V mutation rather than the presence of HLA-B27, elucidating genetic nuances shaping distinct clinical outcomes.

From a demographic perspective, it is well-established that axSpA, particularly AS, tends to affect males more prominently, while FMF shows an equal distribution between sexes.^[1,2]^ However, various studies have indicated varying trends in the sex distribution of FMF-SpA cases.^[10,12,13,24]^ This variance could potentially be attributed to methodological differences among studies. In our study, we note a more balanced representation of both sexes.

The reported prevalence of FMF-SpA varies in the literature due to differences in patient cohorts among studies. For instance, within a group of 503 FMF patients, sacroiliitis was detected in 6% of those with amyloidosis and 11% without amyloidosis.^[25]^ Another study involving 201 FMF patients found frequencies of 7.5% for AS and 8.9% for axSpA.^[15]^ In a separate investigation of 283 FMF patients, 26.1% were diagnosed with FMF-SpA. Within this group, 86% were classified as axSpA, while the rest fell into the category of peripheral SpA.^[12]^ In a cohort of 136 patients with both FMF and axSpA, the majority (84%) had AS.^[24]^ In a larger FMF patient cohort encompassing 971 individuals, an in-depth examination of associated inflammatory diseases with FMF showed that the most prevalent inflammatory condition was SpA, with a prevalence of 12.9%.^[26]^ In contrast to previous studies, which often focused on specific FMF-associated clinical manifestations such as sacroiliitis within the FMF cohort, we exclusively included patients with confirmed diagnoses of both FMF and SpA. In our cohort, axSpA was the predominant SpA classification, with nearly 80% of patients receiving an AS diagnosis. These findings, in conjunction with the data mentioned earlier, underscore the prominence of axial symptoms as key clinical manifestations of SpA rather than peripheral symptoms and highlight that a significant proportion of patients are diagnosed with AS. However, it’s important to acknowledge the potential for bias in this context, as milder forms of SpA without radiographic evidence might receive less attention, and arthritis could be attributed to FMF rather than SpA, leading to bias and underestimation of peripheral SpA.

Peripheral arthritis is a prominent feature of SpA, frequently affecting the large joints of the lower extremities in a chronic and oligo-articular pattern. Similarly, arthritis in FMF primarily targets the lower extremities, displaying an intermittent involvement of large joints. This tendency leans towards a mono-oligoarticular presentation, with some patients transitioning into a chronic and potentially destructive phase. Notably, this destructive progression is particularly evident in the hip joints.^[1]^ Within studies focusing on FMF-SpA cohorts, it has been observed that the prevalence of arthritis stands at approximately 40%.^[11,12,24]^ Given the similarity in the presentation and pattern of arthritis between the 2 conditions, it becomes challenging to attribute the cause of arthritis to one specific disease definitively. In our study, the frequency of peripheral arthritis was notably higher at about 70%, with the majority of cases exhibiting intermittent involvement and affecting large joints. Approximately one-quarter of the patients with peripheral arthritis experienced chronic arthritis.

Hip joint involvement is observed in approximately 40% of patients with AS, with nearly 5% of cases necessitating arthroplasty.^[27]^ However, the association between HLA-B27 and hip joint disease remains contradictory.^[28]^ In the case of FMF, hip involvement is considered a manifestation of chronic arthritis, and the exact prevalence of this condition remains uncertain. In a recent study, approximately 20% of FMF-SpA patients exhibited moderate to severe hip disease, with a hip replacement rate of around 10%. Notably, M694V was prevalent among patients with hip involvement.^[24]^ Our own study revealed that 15% of patients exhibited radiologically confirmed moderate-to-severe hip involvement, and 8.7% underwent a hip replacement, consistent with previous findings. Additionally, we observed a significant association between hip involvement and M694V, while HLA-B27 did not appear to be linked to hip involvement.

HLA-B27 stands as a hallmark feature of SpA, with a prevalence of approximately 90% in Western populations.^[29]^ Notably, studies have revealed that B27 positivity in about 70% of adults with AS in Turkish patients.^[29]^ HLA-B27 positivity is as low as 30% in some SpA subtypes.^[30]^ However, in the context of FMF-SpA, the reported prevalence of HLA-B27 is notably lower, ranging between 0% and 40%.^[10,15,24]^ In our study, HLA-B27 results were available for 106 FMF-SpA patients, and HLA-B27 was observed in 30% of them. In the current study, no significant distinctions in HLA-B27 positivity were noted concerning FMF features like fever and serositis or SpA-related findings like uveitis, enthesitis, and mNY grade sacroiliitis. However, a significant association was observed between amyloidosis and HLA-B27.

In this study, we noted a substantial predominance of M694V variant, accounting for 60.4% of alleles and 85.2% of carriers. Notably, the association between AS and the M694V mutation holds particular importance for patients who lack the HLA-B27 gene,^[31]^ further emphasizing the potential impact of MEFV-related autoinflammatory pathways in the pathogenesis of SpA.^[3,31]^

The impact of MEFV mutations on clinical features in AS is currently not well understood, and existing data present contradictions regarding whether MEFV contributes to a more severe phenotype.^[32]^ However, it has been reported that serum IL-1β and IL-23 levels were significantly elevated in AS patients carrying the MEFV 694V variant,^[3]^ suggesting a potential pathogenic abnormality at the molecular level.

In FMF-associated spondylitis, there is limited available data regarding the influence of MEFV mutations on clinical symptoms. To date, only one study has explored the impact of the M694V mutation on clinical and radiological features.^[24]^ According to this study, patients with the M694V mutation exhibited a higher prevalence of erysipelas-like erythema, increased rates of peripheral arthritis, and a greater likelihood of hip involvement. In our own study, we found that the presence of any MEFV mutations was associated with hip joint involvement. Furthermore, when we specifically analyzed patients with the M694V mutation, we observed significant associations with erysipelas-like erythema, chronic arthritis, hip involvement, the need for hip prostheses, and the development of amyloidosis. Interestingly, there was a trend suggesting that dactylitis was less frequently observed in individuals with M694V mutations. Considering the potentially severe outcomes such as chronic arthritis, hip involvement, and amyloidosis, our findings suggest that the presence of M694V mutations may further emphasize a predisposition to a severe disease phenotype.

New bone formation, a defining characteristic of SpA, is often indicated by the presence of syndesmophytes. In AS patients, syndesmophytes are typically observed in approximately 40% of cases when the disease has persisted for about ten years. This incidence increases to about 60% in patients with a mean disease duration of roughly 20 years at the time of diagnosis.^[33]^ There is a limited number of studies that have assessed vertebral damage in SpA-FMF patients. In a recent study, approximately 40% and 20% of the patients exhibited cervical and lumbar syndesmophytes, respectively, and 5% of the group displayed the characteristic bamboo spine appearance. Notably, the study did not report the disease duration of the SpA patients. Comparatively, these frequencies did not significantly differ between SpA-FMF patients and the axSpA group.^[24]^ In our study, we made similar observations, finding that around 40% of our patients showed varying degrees of spinal involvement. This observation aligns with the expected prevalence based on a median disease duration of approximately ten years. However, it’s worth noting that neither the presence of M694V nor HLA-B27 was associated with the presence of syndesmophytes in our study. As a limitation, we should mention that we did not quantitatively score the extent of spinal damage; instead, we categorized the presence or absence of syndesmophytes.

Amyloidosis is a significant concern in FMF, with an estimated occurrence rate of 9% among FMF patients.^[34]^ Similarly, it poses a concern in AS, where its prevalence is reported to be approximately 1%.^[35]^ Notably, the M694V genotype exhibits a significant correlation with the development of amyloidosis in FMF.^[34]^ In our study, we observed that 10% of the patients had documented amyloidosis. When compared with the existing literature, it became evident that this condition is more prevalent among individuals carrying the M694V mutation. Intriguingly, HLA-B27 also exhibits an association with amyloidosis, and patients testing positive for HLA-B27 tend to have a higher incidence of amyloidosis. In our regression analysis, both the M694V mutation and HLA-B27 were predictive factors for the development of amyloidosis.

While our study has yielded valuable findings, we must acknowledge several limitations. The retrospective nature of our research may have introduced selection bias and hindered comprehensive data collection. It’s important to note that we did not specifically analyze the impact of medications on disease activity for each condition, leaving this as a subject for future investigations. Additionally, the absence of universal HLA-B27 testing and imaging modalities, such as conventional radiographs and sacroiliac magnetic resonance imaging, may have affected the assessment of subgroups within SpA. The relatively modest sample size may also restrict the generalizability of our results to a broader population. Furthermore, our study primarily focused on the Turkish people, and it’s essential to consider that ethnic and genetic variations could influence the observed outcomes.

In summary, our study is significant in multiple ways despite certain limitations. FMF-AS is a rare condition with limited research, making our study one of the largest cohorts to date on this association. Our work enriches the existing knowledge base by offering a deeper understanding of this uncommon condition. Additionally, our research goes beyond simple clinical observation by examining both radiological severity and the impact of MEFV mutations and HLA-B27 on clinical manifestations. Future studies are needed to confirm our findings and to further investigate the role of MEFV mutations, HLA-B27, on the disease severity, clinical manifestations, and prognosis of this rare condition.

## Author contributions

**Conceptualization:** Aslihan Avanoglu Guler, Nazife Sule Yasar Bilge, Timucin Kasifoglu, Abdurrahman Tufan, Ismail Sari.

**Data curation:** Tuba Yuce Inel, Aslihan Avanoglu Guler, Nazife Sule Yasar Bilge.

**Formal analysis:** Tuba Yuce Inel, Abdurrahman Tufan, Ismail Sari.

**Investigation:** Tuba Yuce Inel, Aslihan Avanoglu Guler, Nazife Sule Yasar Bilge, Ismail Sari.

**Methodology:** Tuba Yuce Inel, Timucin Kasifoglu, Abdurrahman Tufan.

**Resources:** Tuba Yuce Inel, Nazife Sule Yasar Bilge.

**Supervision:** Timucin Kasifoglu, Abdurrahman Tufan, Ismail Sari.

**Writing – original draft:** Tuba Yuce Ine.

**Writing – review & editing:** Timucin Kasifoglu, Abdurrahman Tufan, Ismail Sari.

## Supplementary Material


