# Supplementary material for: Effect of MEFV mutations and HLA-B27 on clinical findings of familial Mediterranean fever and spondyloarthritis
Source: Medicine (Baltimore). 2025 Oct 24;104(43):e45344. doi: 10.1097/MD.0000000000045344 (PMC12558185; doi:10.1097/MD.0000000000045344)
Supplement: Supplementary file 1 [file medi-104-e45344-s001.docx]

| **Supplementary Table-1. Distributions of MEFV gene variations in FMF-SpA patients** | | |  |
| --- | --- | --- | --- |
| Any MEFV allele frequency, n (%) | 202 (87.8) | |  |
| M694V | 139 (60.4) | |  |
| M680I | 23 (10) | |  |
| V726A | 11 (4.8) | |  |
| E148Q | 6 (2.6) | |  |
| R202Q | 14 (6.1) | |  |
| P369S | | 3 (1.3) | |
| A744S | 3 (1.3) | |  |
| R761H | 3 (1.3) | |  |

| **Rate of exon 10 mutations, n (%)** | |
| --- | --- |
| M694V homozygous | 52 (51.0) |
| M694V heterozygous | 19 (18.6) |
| M694V/M680I compound heterozygous | 11 (10.8) |
| M680I/V726A compound heterozygous | 4 (3.9) |
| V726A heterozygous | 4 (3.9) |
| M694V/V726A compound heterozygous | 3 (2.9) |
| M680I heterozygous | 3 (2.9) |
| M680I homozygous | 2 (2.0) |
| M694V/R761H compound heterozygous | 1 (1.0) |
| R761H homozygous | 1 (1.0) |
| M694V/A744S compound heterozygous | 1 (1.0) |
| A744S heterozygous | 1 (1.0) |

| **Distribution of homozygous or compound heterozygous mutations in exon 10, n (%)** | |
| --- | --- |
| M694V homozygous | 52 (69.3) |
| M694V/M680I | 11 (14.7) |
| M680I/V726A | 4 (5.3) |
| M694V/V726A | 3 (4.0) |
| M680I homozygous | 2 (2.7) |
| M694V/R761H | 1 (1.3) |
| R761H homozygous | 1 (1.3) |
| M694V/A744S | 1 (1.3) |

| **Supplemental Table 2. The effect of MEFV mutation on clinical findings of FMF and SpA** | | | |
| --- | --- | --- | --- |
|  | **MEFV mutation (+)**  **n (%)** | **MEFV mutation (-)**  **n (%)** | **p-value** |
| **Gender (male)** | 106 (52.5) | 12 (42.9) | 0.340 |
| **FMF clinical findings** |  |  |  |
| Fever | 170 (84.2) | 24 (85.7) | 0.545 |
| Peritonitis | 173 (85.6) | 23 (82.1) | 0.400 |
| Pleuritis | 106 (52.5) | 16 (57.1) | 0.643 |
| Erysipelas-like rash | 70 (34.7) | 8 (28.6) | 0.524 |
| Febrile myalgia | 53 (26.7) | 5 (17.9) | 0.339 |
| **The current dominant SpA clinic**  Peripheral dominant  Axial dominant | 37 (18.3)  165 (81.7) | 9 (32.1)  19 (67.9) | 0.087 |
| **SpA clinical findings** |  |  |  |
| Inflammatory back pain | 170 (84.2) | 24 (85.7) | 0.55 |
| Enthesitis | 34 (16.8) | 6 (21.4) | 0.35 |
| Dactylitis | 6 (3) | 2 (7.1) | 0.25 |
| Uveitis | 13 (6.4) | 1 (3.6) | 0.47 |
| Inflammatory bowel disease | 19 (9.5) | 1 (3.6) | 0.26 |
| Psoriasis | 5 (2.5) | 1 (3.6) | 0.55 |
| **Chronic arthritis** | 58 (29.0) | 6 (21.4) | 0.4 |
| **Arthritis type**  Monoarthritis  Oligoarthritis | 22 (16.2)  114 (83.8) | 2 (14.3)  12 (85.7) | 0.61 |
| **Arthritis pattern**  Intermittent  Additive  Migratory | 131 (89.7)  3 (2.1)  12 (8.2) | 15 (83.3)  1 (5.6)  2 (11.1) | 0.6 |
| **HLA-B27 positivity** | 43 (26.4) | 9 (39.1) | 0.2 |
| **Amyloidosis** | 22 (10.9) | 2 (7.1) | 0.42 |
| **Any syndesmophyte** | 73 (41.2) | 11 (44) | 0.79 |
| **Cervical+lumbar syndesmophyte** | 31 (19) | 5 (21.7) | 0.47 |
| **Moderate to severe hip joint involvement** | 60 (31.4) | 4 (14.8) | 0.08 |
| **Hip prosthesis** | 22 (10.9) | 0 (0) | 0.04 |
| Please be aware that the analysis was performed within the tested patient group. As a result, the numerical outcomes deviate from the actual counts based on the number of individuals. This discrepancy arises because the numbers presented represent allelic counts, not individual counts. | | | |
